# A serious thrombotic event in a patient with immune thrombocytopenia requiring intravenous immunoglobulin: a case report

**DOI:** 10.1186/s13256-018-1955-x

**Published:** 2019-01-28

**Authors:** Tarinee Rungjirajittranon, Weerapat Owattanapanich

**Affiliations:** 1 Division of Medicine, Phranangklao Hospital, Nonthaburi, Thailand; 2 Division of Hematology, Department of Medicine, Faculty of Medicine Siriraj Hospital, Mahidol University, Bangkok, Thailand

**Keywords:** Immune thrombocytopenia, Thrombosis, Stroke, Intravenous immunoglobulin

## Abstract

**Background:**

Immune thrombocytopenia is an acquired autoimmune disease. Recently, there has been evidence of thrombotic risk in patients with immune thrombocytopenia, but the mechanism is still inconclusive. Intravenous immunoglobulin infusion therapy is considered an efficient treatment; however, it still is associated with adverse events of fever, chills, and hypotension, as well as serious complications such as thrombosis. We report a case a patient with relapsed immune thrombocytopenia who developed ischemic stroke after an intravenous immunoglobulin infusion.

**Case presentation:**

A 49-year-old Thai woman with relapsed/refractory immune thrombocytopenia came to our hospital with a large hematoma at the right buttock, and her platelet was decreased to 3 × 10^9^/L. She was admitted to our hospital for intravenous immunoglobulin administration. One hour after completion of intravenous immunoglobulin infusion, the patient’s sister complained that the patient was unconscious and could not move both legs and arms. Emergency computed tomography of the brain showed no abnormal findings, such as brain edema, intracranial hemorrhage, or infarction. One day later, repeat computed tomography of the brain displayed extensive acute ischemic changes and loss of gray-white differentiation of bilateral cerebral hemispheres.

**Conclusions:**

We performed an extensive literature review to determine the possible causes of serious thrombotic events in immune thrombocytopenia between the predictive factors of the disease and intravenous immunoglobulin. Although intravenous immunoglobulin is an effective treatment, thrombotic complications can occur. We emphasize that in patients with atherosclerosis risk factors or thrombophilia, the appropriateness of administering an intravenous immunoglobulin infusion should be carefully evaluated.

**Electronic supplementary material:**

The online version of this article (https://doi.org/10.1186/s13256-018-1955-x) contains supplementary material, which is available to authorized users.

## Background

Immune thrombocytopenia (ITP) is an acquired autoimmune disease that results in a low platelet count of less than 100 × 10^9^/L. The incidence of primary ITP among adults is 3.3 per 100,000 adults per year [1]. The pathophysiology of this condition is associated with increased platelet destruction and impaired megakaryocyte production [2, 3]. A recent meta-analysis revealed that the annualized cumulative thrombosis risk among adult patients with ITP was 3% per year [4]. However, the possible mechanisms of the thromboembolic events are multifactorial and inconclusive. According to the mechanism of ITP, there are several treatment options, including corticosteroids, immunosuppressive agents, anti-D, thrombopoietin agonists, splenectomy, and intravenous immunoglobulin (IVIg). IVIg is usually used for first-line treatment, especially in emergency conditions. The mechanism of action is to saturate the Fc receptors in the reticuloendothelial system, leading to a decreased destruction of platelets [5]. Although IVIg is considered a safe treatment, it still is associated with adverse events, namely fever, chills, and hypotension, in addition to more serious complications, such as deep venous thrombosis, pulmonary embolism, and arterial thrombosis [6, 7]. We report a case of a patient with active ITP who developed an acute stroke following IVIg administration.

Several incidents of thrombosis developing after IVIg treatment have been reported in patients with neurological disorders. The patients had no previous history of a bleeding tendency [8–11]. However, our patient with ITP had active bleeding and then developed an acute stroke following IVIg administration. This case represents an example of a serious arterial thrombotic event that can occur after ITP treatment. The pathophysiologies of thrombosis in ITP itself and in medication-associated thrombosis were extensively reviewed and are discussed.

## Case presentation

A 49-year-old Thai woman with relapsed/refractory ITP was diagnosed in December 2016 with petechiae on her legs. She was a shop owner in Nonthaburi Province, Thailand. Her complete blood count (CBC) showed hemoglobin of 13 g/dl, a white blood cell count of 7 × 10^9^/L, and a platelet count of 4 × 10^9^/L. The results of her urinalysis and renal and liver function tests were normal. The results of all of her other blood tests (including viral hepatitis, anti-human immunodeficiency virus, and antiphospholipid profiles) were negative. She also had underlying diseases of poorly controlled diabetes mellitus type 2, hypertension, and hyperthyroidism. She denied having any other medical illness or a history of surgery. Her first-degree family members were healthy and had no history of hematological disorders. She had no history of smoking or alcohol consumption. Her current medications were losartan 100 mg/day, metformin 2000 mg/day, glipizide 20 mg/day, pioglitazone 30 mg/day, atorvastatin 40 mg/day, and methimazole 5 mg/day.

Her platelet count responded well to the normal range with oral prednisolone, and the prednisolone was tapered in January 2017. The first relapse episode happened in August 2017. She presented with bleeding from the gums, and treatment was reinitiated with steroids. Once her CBC was normal, the treatment was gradually tapered. The last event occurred in October 2017, when her platelet count dropped to 36 × 10^9^/L without clinical bleeding. After treatment with high-dose prednisolone for 1 month, her platelet count recovered to the normal range. Although the prednisolone dosage was decreased gradually by 10 mg per week, she could not maintain her platelet count with prednisolone 0.5 mg/kg/day. She was therefore treated with 50 mg/day of azathioprine and 200 mg/day of danazol, combined with a high-dose prednisolone, to increase her platelet count.

**Fig. 1:**
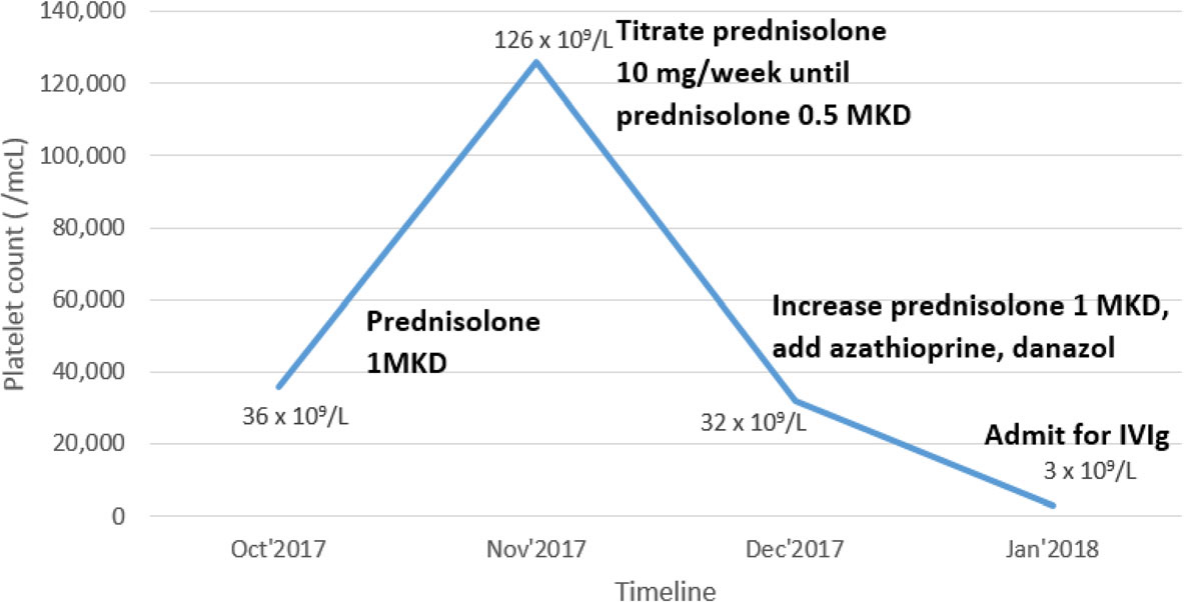
Timeline of treatments and platelet counts of the patient. Abbreviations: *Dec* December, *IVIg* intravenous immunoglobulin, *Jan* January, *L* liter, *mcL* microliter, *MKD* mg/kg/day, *Nov* November, *Oct* October

**Fig. 2:**
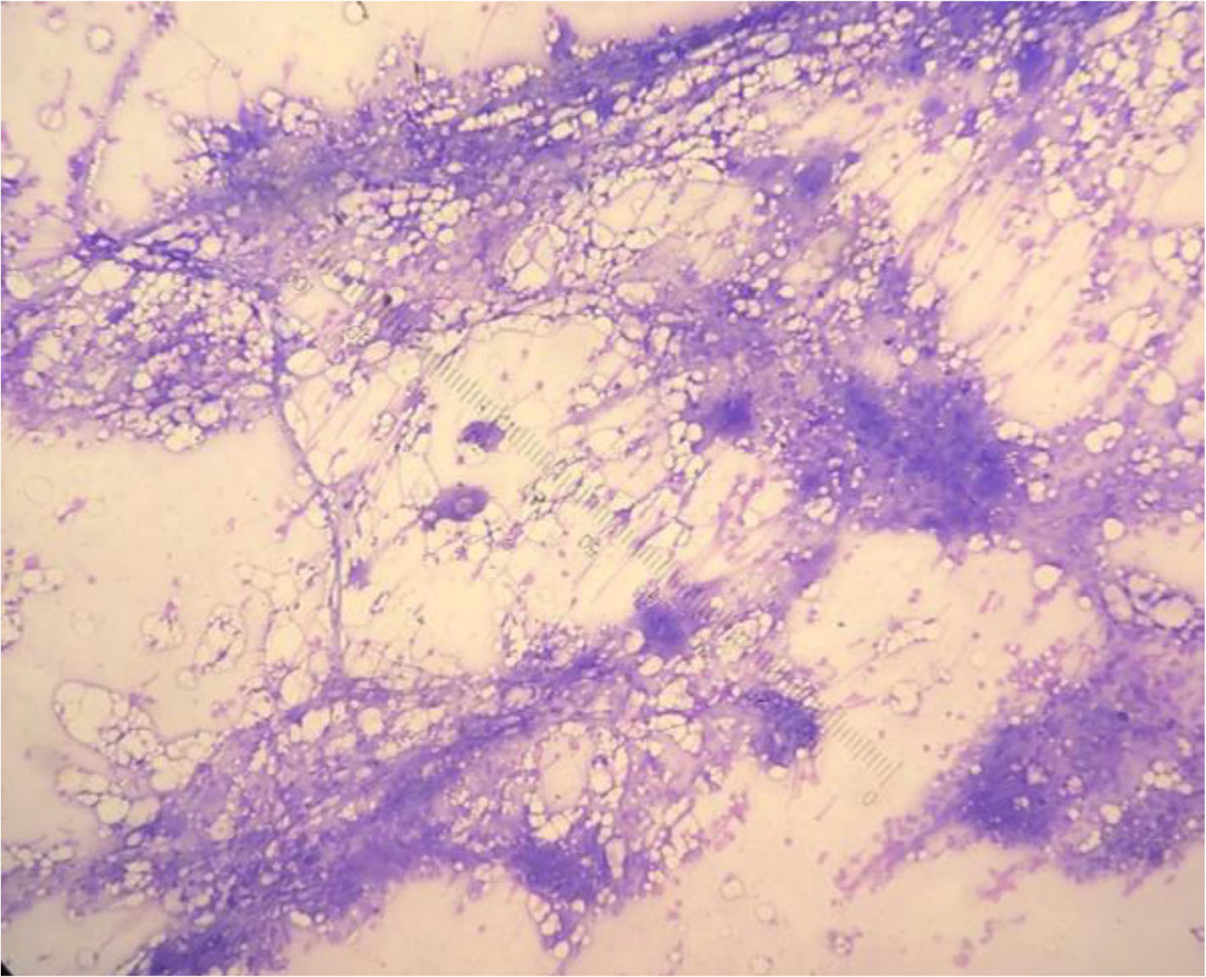
Bone marrow aspiration demonstrating increased number of megakaryocytes

In January 2018, she came to our hospital with a large hematoma on her right buttock. Her initial vital signs showed a temperature of 37.2 °C, pulse rate of 87/minute, respiratory rate of 14/minute, and blood pressure of 125/82 mmHg. The results of her physical examinations (cardiovascular, respiratory, gastrointestinal, and neurological) were normal, except for the presence of a large hematoma about 10 cm in diameter on her right buttock. Her CBC showed hemoglobin of 11.5 g/dl, a white blood cell count of 10.4 × 10^9^/L, and a decreased platelet count of 3 × 10^9^/L. Other initial laboratory findings (including a renal function test, liver function test, and urinalysis) were found to be within normal limits. She was admitted to the hospital for IVIg administration. The timelines of her treatments and her platelet counts are illustrated in Fig. 1. A bone marrow study was performed, which revealed an increase in the number of megakaryocytes, compatible with peripheral destruction (Fig. 2). Intravenous dexamethasone (40 mg/day) and IVIg 60 g/day (1 g/kg/day) were initiated. The infusion rate of IVIg was 40 ml/hour for 1 hour and then 60 ml/hour. The patient was also given premedication (4 mg of intravenous chlorpheniramine).

**Fig. 3:**
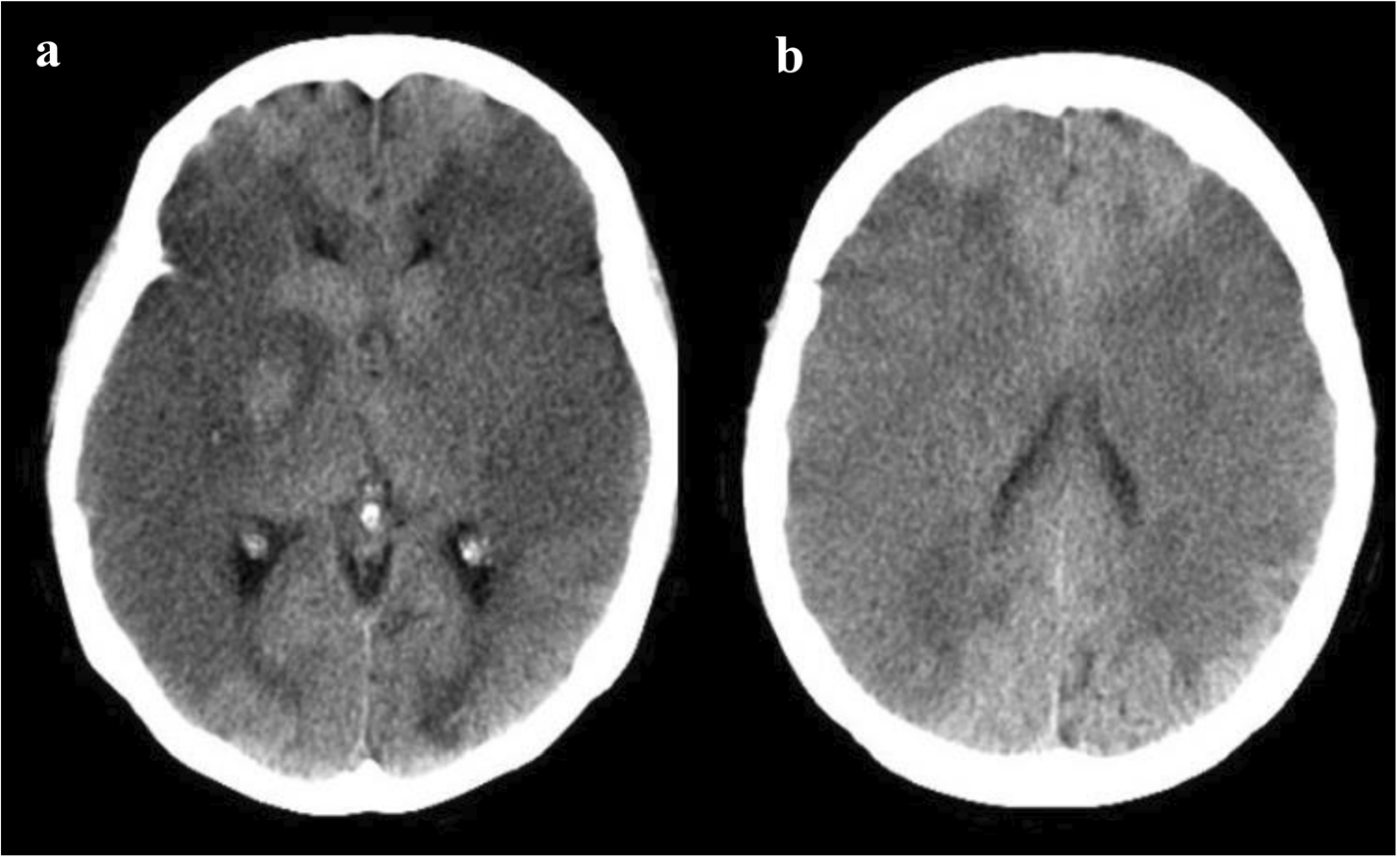
Computed tomography of the brain. **a** Decrease in attenuation and loss of gray-white differentiation of bilateral cerebral hemispheres supplied by the middle cerebral artery territories, with a narrowing of the bilateral lateral ventricle due to compression by swelling brain parenchyma. **b** The infarction in the right and left middle cerebral artery distribution, with sparing of the bilateral frontal and occipital lobes

One hour after completion of the IVIg infusion, the patient’s sister complained that the patient was unconscious and had not been able to move both legs and arms. A neurological examination showed a Glasgow Coma Scale score of E3V3M5 and motor power of grade 2 on both sides; both pupils were 5 mm and semireactive to lights. Emergency computed tomography (CT) of the brain showed no abnormal findings, such as brain edema, intracranial hemorrhage, or infarction. One day later, repeat CT of the brain displayed extensive acute ischemic changes and loss of gray-white differentiation of bilateral cerebral hemispheres (Fig. 3). The patient’s consciousness was deteriorating. The decision was made to forgo intubation at the request of the family and in accordance with the patient’s advance care directive. Consequently, her blood pressure dropped rapidly, and she died within a few hours. Her family members declined an autopsy. A timeline of the long-term treatment of the patient is provided in Additional file 1.

## Discussion

We present a case of a 49-year-old woman with relapsed/refractory ITP who had severe thrombocytopenia and a large hematoma on her right buttock. After IVIg therapy for active ITP, she unexpectedly developed acute ischemic stroke. Many case reports have described thrombotic events after IVIg administration, including venous and arterial thrombosis [8–11]. The incidence rate of post-IVIg thrombosis varies between 0.6% and 3% of patients. Our literature review revealed that IVIg-related arterial thromboses are more common than venous thromboses, including myocardial infarction, stroke, and peripheral arterial disease [6, 12]. Focusing on the several reports available on ischemic stroke occurrence after IVIg therapy, we found five cases in all, including our patient’s [8–10, 13]. All previous cases had no underlying bleeding disorders and had normal platelet counts. Interestingly, only our patient had very low platelet numbers with a recently developed bleeding history, but she had an ischemic stroke after IVIg therapy. In contrast to the four other cases, our patient also had atherosclerosis risks, including diabetes mellitus type 2 and hypertension, which might be possible additive risks for thrombosis. Furthermore, it seemed that she received a higher daily dose of IVIg than the patients in the previous reports. Details of the reported patients who had an ischemic stroke after IVIg therapy are provided in Table 1.

**Table 1:** Reported cases with development of ischemic stroke after intravenous immunoglobulin therapy

References	Age/sex	Underlying disease	Baseline platelet count (×10^9^/L)	Indication for IVIg therapy	IVIg dosage	Duration between time of IVIg initiation and ischemic stroke occurrence	Brain imaging findings	Outcomes
Sztajzel 1999 [10]	46-year-old female	Guillain-Barré syndrome	NR	Severe polyradiculoneuropathy	0.4 g/kg/day for 5 days	5 days	Bilateral hypodensities in the capsulolenticular regions	Motor full recovery, but memory difficulties and impairment of the executive functions
Alexandrescu 2005 [9]	82-year-old male	CIDP	190	Monthly IVIg administration for CIDP treatment (previously treated with IVIg of 86 doses without any adverse reactions)	50 g, single dose	Several hours	Large frontoparietal infarct involving the white and gray matter and basal ganglia, without hemorrhage	Partial recovery
Milani 2009 [8]	55-year-old male	Chronic lymphocytic leukemia	170	Hypogammaglobulinemia (previously tolerated multiple infusions of IVIg without any adverse reactions)	0.4 g/kg (30 g), single dose	12 hours	Cerebral infarcts seen within the left posterior middle cerebral artery distribution, bilateral high parietal loops, and bilateral occipital lobes	Deceased
Chang 2014 [13]	44-year-old male	Miller Fisher syndrome	NR	Miller Fisher syndrome treatment	0.4 g/kg/day for 5 days, total 180 g	3 weeks	Acute left parieto-occipital infarct with hemorrhagic transformation and perilesional edema	Partial recovery
Our patient	49-year-old female	ITP, DM, HT, hyperthyroidism	3	Active ITP with large hematoma at right buttock	1 g/kg/day (60 g), single dose	12 hours	Extensive acute ischemic changed and hemorrhagic transformation in bilateral asymmetrical cerebral white matter as well as left basal ganglion and cerebral peduncle	Deceased

*Abbreviations: CIDP* Chronic inflammatory demyelinating polyneuropathy, *DM* Diabetes mellitus, *HT* Hypertension, *ITP* Immune thrombocytopenia, *IVIg* Intravenous immunoglobulin, *NR* Not reported

The onset time of IVIg-related thrombotic complications is variable. In one retrospective series, half of the patients who exhibited thrombotic complications developed the complications during the IVIg infusion, with a further 33% developing during days 1 and 2 and 17% developing on day 8 after the IVIg infusion [12]. Several mechanisms of thrombosis have been proposed in the literature [6, 14]. Thrombotic events may be caused by increased serum viscosity exceeding the upper limit of normal, especially up to fourfold higher, thereby acting synergistically with albumin and fibrinogen to promote blood cell aggregation [12, 14, 15]. Consequently, it is important to increase IVIg infusion rates gradually. In addition, an IVIg infusion may induce platelet aggregation activity by enhancing adenosine triphosphate release [12, 16]. Moreover, the infusion may have a procoagulant activity via activated factor XI [12, 17]. Last, the IVIg infusion may alter cytokine synthesis, especially that of the vasoconstrictive cytokines, resulting in arterial vasospasm [12, 17–19].

Apart from the side effects of IVIg treatment, there is much evidence that the thrombotic complications are related to the ITP itself. In population-based studies [20–22], there is a trend for an increased risk of arterial thrombosis with chronic ITP, but statistical significance mostly is not reached. Approximately one-fifth of patients with chronic ITP were found to have an increase in microparticles. Inside the microparticles are procoagulant materials that can induce the coagulation pathway [16–18]. Moreover, those patients also had higher levels of factors VIII, IX, and XI than the control subjects. Nitric oxide depletion due to continuous immune activation has been suggested as one thrombotic complication. Indeed, atherosclerotic risk factors can also contribute to arterial thrombosis, especially in our patient, given that she had poorly controlled diabetes mellitus type 2. We suggest that for those patients who have other thrombotic risks such as diabetes or antiphospholipid syndrome, the appropriateness of administering an IVIg infusion should be very carefully considered. Hydration and a slow infusion rate may reduce the risk of hyperviscosity during an IVIg infusion.

## Conclusions

In addition to the bleeding manifestations that are commonly found in patients with ITP, several pathogeneses of thrombosis were also documented in our patient. Although IVIg is an effective treatment, especially in the case of life-threatening bleeding in a patient with ITP, the possibility of thrombotic complications must not be overlooked, because patients with ITP have their own thrombotic risk factors. In patients with ITP who have an underlying disease with an atherosclerotic risk or thrombophilia, prescription of an IVIg should be carefully considered. In the event of IVIg administration, such patients should subsequently be observed for thrombotic complications. The IVIg infusion rate and daily dose need to be further investigated to determine whether they have any impact on the risk of thrombosis.

## Additional file

Additional file 1: A timeline of the long-term treatment of the patient. (DOCX 18 kb)

## Abbreviations

CBC: Complete blood count
CIDP: Chronic inflammatory demyelinating polyneuropathy
CT: Computed tomography
DM: Diabetes mellitus
HT: Hypertension
ITP: Immune thrombocytopenia
IVIg: Intravenous immunoglobulin
