# Supplementary material for: A serious thrombotic event in a patient with immune thrombocytopenia requiring intravenous immunoglobulin: a case report
Source: J Med Case Rep. 2019 Jan 28;13:25. doi: 10.1186/s13256-018-1955-x (PMC6348674; doi:10.1186/s13256-018-1955-x)
Supplement: Supplementary file 1 — A timeline of the long-term treatment of the patient. (DOCX 18 kb) [file 13256_2018_1955_MOESM1_ESM.docx]

| **Dates** | **Relevant Past Medical History and Interventions** | | |
| --- | --- | --- | --- |
| Since 2010 | She also had diabetes mellitus type 2, hypertension, and hyperthyroidism. She had poor- controlled blood sugar. Blood test for HbA1c was 12% (8 Oct 2017). | | |
| **Dates** | **Summaries from Initial and Follow-up Visits** | **Diagnostic Testings**  **(including dates)** | **Interventions** |
| 6 Dec 2016 | She noticed petechiae on both legs. She had no other bleeding symptoms. Her treatment was started with oral prednisolone with good response. Prednisolone was taper off in January 2017 | The investigations for diagnosis of Immune thrombocytopenia consisted of:   - CBC: Hb 13 g/dL, WBC 7 x 10^9^/L, Platelet 4 x 10^9^/L   (6Dec2016)   - Viral hepatitis profiles: negative (6Dec2016) - Anti-HIV: negative   (6Dec2016)   - Antiphospholipid profile: negative   (11Dec2016)   - Lupus anticoagulant: negative   (11Dec2016) | - None |
| 22 Aug 2017 | Her first relapse episode happened. She had spontaneous bleeding per gums. Treatment was re-initiated with prednisolone 1 mg/kg/day. At this time, treatment was complete response and gradually withheld prednisolone in September 2017. | The investigations for diagnosis of relapsed Immune thrombocytopenia consisted of:   - CBC: Hb 12 g/dL, WBC 9 x 10^9^/L, Platelet 10 x 10^9^/L   (22Dec2017) | - None |
| 31 Oct 2017 | Second relapsed of ITP was occurred in Oct 2017 during her follow up, this time platelet count was dropped to 36 x 10^9^/L. She had no clinical bleeding. After treatment with high dose prednisolone for a month, platelet count was recovery to normal range. Therefore, the prednisolone dosage was gradually decreased by 10 mg per week, but she could not maintain her platelet number at prednisolone 0.5 MKD. Therefore, she was treated with 50 mg/day of azathioprine and 200 mg/day of danazol combined with high dose prednisolone to increase platelet count(at least 40 mg/day). | The investigations for diagnosis of relapsed Immune thrombocytopenia consisted of:   - CBC: Hb 12 g/dL, WBC 6 x 10^9^/L, Platelet 36 x 10^9^/L   (31Oct2017) | - None |
| 20 Jan 2018 | She presented to the hospital with large hematoma at right buttock. She was admitted in the hospital.  One hour after IVIG complete infusion, she had neurological deterioration. | - CBC: Hb 10 g/dL, WBC 10 x 10^9^/L, Platelet 3 x 10^9^/L   (20Jan2018)   - Bone marrow study   (20Jan2018): The finding displayed increasing in number of megakaryocytes compatible with peripheral destruction   - Dexamethasone 40 mg/day (start 20Jan2018) - IVIg (start 20-21 Jan 2018) | -Bone marrow study  (20Jan2018)  -IVIg 1 gm/kg/day for 2 days (20-21Jan2018), the infusion rate of IVIg was 40 mL/hour for an hour then 60 mL/hour. Intravenous chlorpheniramine was given for pre-medication.  -CT brain(non-contrast)  (21Jan2018)  -Repeated CT brain  (22Jan2018) |
